# A Case Report of Takayasu’s Arteritis With Cerebral Infarction As Initial Presentation

**DOI:** 10.7759/cureus.30472

**Published:** 2022-10-19

**Authors:** Tehsim Memon, Tufayl Ahmed M Shekha, Pavan Acharya, Rifath I Nishu, Naila Akhter

**Affiliations:** 1 Internal Medicine, Smt. Nathiba Hargovandas Lakhmichand (NHL) Municipal Medical College, Ahmedabad, IND; 2 Internal Medicine, Rajarajeswari Medical College and Hospital, Bengaluru, IND; 3 General Surgery, Shahabuddin Medical College and Hospital, Dhaka, BGD; 4 Internal Medicine, Bangladesh Medical College and Hospital, Dhaka, BGD

**Keywords:** takayasu arteritits, young onset stroke, large vessel vasculitis, : aortitis, pulseless disease

## Abstract

Takayasu’s arteritis is a chronic inflammation of the large arteries such as the aorta and its primary branches, causing progressive arterial occlusion. This leads to reduced blood flow in the limbs and organs, resulting in arm or leg claudication, diminished or absent peripheral pulses, and end-organ ischemia. Stroke is one of the common complications; however, it is rarely the initial presentation. We describe one such case of a 16-year-old female, who presented with right-sided hemiparesis and non-fluent aphasia, without any significant past history. On examination, her right arm was cold and pulseless. She was extensively investigated for the cause of her presentation. Only non-specific inflammatory markers such as erythrocyte sedimentation rate (ESR) were elevated. Imaging studies revealed left middle cerebral artery territory infarct with occlusion of common carotid arteries, bilateral bifurcation, most parts of the left internal carotid artery, and the proximal part of the right internal carotid artery. She was diagnosed with Takayasu's arteritis and was prescribed steroids, on which she gradually recovered and was discharged. In conclusion, young patients, who present with stroke, should be investigated for Takayasu’s arteritis, which leads to earlier treatment and prevention of further life-threatening end-organ damage.

## Introduction

Takayasu’s arteritis is characterized by chronic granulomatous inflammation of the large arteries such as the aorta and its primary branches, causing narrowing, occlusion, dilatation, or aneurysms of the vessels [1]. Its presentation is characterized by two stages, with an early, sub-acute, “pre-pulseless” stage presenting as non-specific constitutional symptoms, such as low-grade fever, fatigability, weight loss, arthralgia, and myalgia, followed by a late, chronic, pulseless stage presenting with the symptoms of end-organ ischemia due to stenosis of the vessels. However, this classic presentation does not hold true for all patients [2,3]. In this chronic stage, clinical presentation depends on the arteries involved. The most common presentation is diminished or absent pulses, associated with limb claudication and blood pressure discrepancies [4]. Other manifestations include vascular bruits, particularly affecting the carotids, subclavian, and abdominal vessels, hypertension resulting from renal artery stenosis, Takayasu’s retinopathy, aortic regurgitation resulting from dilatation of the ascending aorta, congestive heart failure due to hypertension, aortic regurgitation and coronary artery disease, neurological features secondary to hypertension and/or ischemia, including seizures, stroke, amaurosis, and pulmonary vasculopathy, leading to pulmonary arterial hypertension [4]. Stroke is a common complication and occurs in 10%-20% of patients [5]. However, stroke as the first manifestation is infrequent and only a few cases are reported [2]. We report one such case, in which the stroke heralded the onset of the disease.

## Case presentation

A 16-year-old girl, without any significant past history, presented to our emergency room with sudden onset of weakness of the right upper limb and lower limb, right facial droop, and difficulty in speaking. She vomited twice on the day of the presentation. Her history was significant for generalized body aches, occasional episodes of arthralgia, and recurrent headaches associated with neck pain, which was radiating to both arms, predominantly on the right side, and was associated with tingling and numbness. On examination, she appeared pale, but she was conscious, oriented, and following verbal commands. On physical examination, her right arm was cold and pulseless, with undetectable blood pressure, while blood pressures on the left arm and both the lower limbs were detectable and normal. Neurological examination revealed non-fluent aphasia with intact comprehension, right-sided dense hemiplegia associated with right-sided supranuclear (central) facial nerve palsy, right-sided tendon jerk hyperreflexia, and extensor plantar response. Laboratory investigations revealed mild hypochromic microcytic anemia, erythrocyte sedimentation rate (ESR) of 60 mm/hour, normal platelet count, blood chemistries, and coagulation profile. Antinuclear antibodies (ANA) and antiphospholipid antibodies (APLA) were negative. Electrocardiogram (ECG), transthoracic echocardiogram (TTE), chest x-ray, and bilateral fundoscopic examination were reported normal.

**Figure 1:**
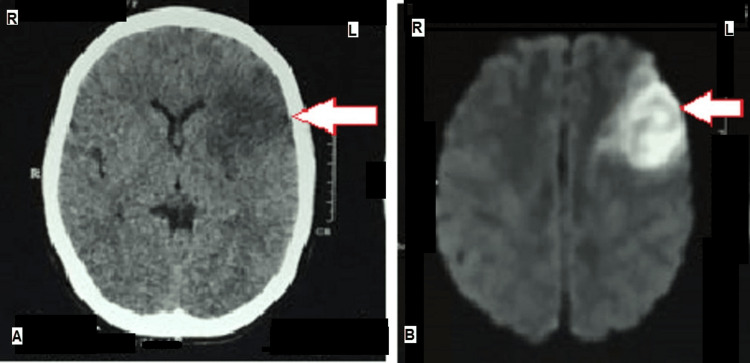
(A) NCCT brain showing left-sided cortical hypodensity (arrow) corresponding to acute infarct. (B) DW-MRI of the brain showing diffusion restriction (hyperintensity) in the left cerebral cortex (arrow) and left gangliocapsular region suggestive of acute non-hemorrhagic infarct. R- Right, L- Left, NCCT- Non-contrast computed tomography, DW-MRI: Diffusion weighted-magnetic resonance imaging

The patient was emergently taken for a non-contrast computed tomography (NCCT) brain, which revealed acute left frontotemporal infarct, without any hemorrhagic transformation (Figure 1A). An urgent neurology consult was obtained and, as the patient was out of the window period for thrombolysis, conservative management was advised. She was prescribed antiplatelet and statin. A brain magnetic resonance imaging (MRI) was performed demonstrating left frontotemporal and left gangliocapsular infarct, with restricted diffusion-weighted signal (hyperintensity) on diffusion-weighted imaging (DWI) (Figure 1B).

**Figure 2:**
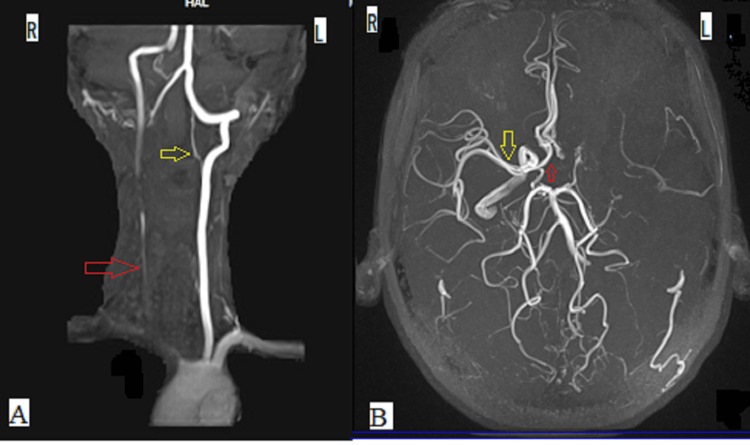
Magnetic resonance angiography (MRA) showing (A) occlusion of the right common carotid artery (red arrow) and left internal carotid artery (yellow arrow), and (B) occlusion of the left middle cerebral artery (MCA) and A1 segment of the left anterior cerebral artery (ACA). A normally enhancing right MCA (yellow arrow) and A1 segment of the right ACA (red arrow) are shown for comparison. R - Right, L - Left

Magnetic resonance angiography (MRA) of the head and neck showed occlusion of the right common carotid artery, bilateral bifurcation, most part of the left internal carotid artery (ICA), left middle cerebral artery (MCA) and its branches, A1 segment of the left anterior cerebral artery (ACA) and intermittent poor flow in the distal part of right ICA and both external carotid arteries (ECA) (Figures 2A, 2B).

In light of the young age of presentation and negative autoimmune profile, a rheumatology consult was obtained and Takayasu’s arteritis was diagnosed according to The American College of Rheumatology criteria 1990 [6].

She was prescribed oral prednisone 40 mg daily, after which she displayed marked clinical improvement and normalization of inflammatory markers by the time of discharge. The same dose of steroids was advised for six weeks and then gradually tapered to 4 mg on alternate days at six months follow-up, without any relapse.

## Discussion

Takayasu arteritis is present worldwide but is more common in Asian countries [3], affecting women more than men, typically presenting between 10 and 40 years [6,7], as seen in our patient, a young, South Asian female, who developed symptoms in her second decade. It has a variable clinical presentation, ranging from asymptomatic disease and constitutional features to symptoms of end-organ damage. Neurological manifestations such as headache, dizziness, syncope, visual impairment, and transient ischemic attacks can occur due to chronic ischemia by progressive stenosis of the common carotid and vertebral arteries. Whereas, stroke can occur due to acute ischemia and infarction by thrombosis or, more commonly, embolism of vessels, which has been reported in 10%-20% of the patients, out of which 80% had anterior circulation involvement [5,8]. However, stroke as the initial presentation has been reported in 5%-8% of the patients only [9]. Thus, the presentation of our patient with acute MCA territory stroke as the first manifestation of Takayasu’s arteritis was rare. Also, her symptoms started suddenly and were maximal at the onset, which along with her MRI findings, indicated that the cerebral infarction was embolic in nature. The source of this embolus can either be cardiac, which was ruled out by the normal TTE, although a transesophageal echocardiogram (TEE) was not done, or it can be from the thrombus in a distant stenosed artery such as common carotid or internal carotid artery, dislodged by the turbulent blood flow, as reported by Kato et al. [10].

While diagnosing Takayuasu’s arteritis has been challenging for a long time, a proper physical examination may help. Patients characteristically present with diminished or absent pulses (most commonly at the level of the radial arteries and are often asymmetric), blood pressure discrepancies and vascular bruits over carotid and subclavian vessels in more than 90% of the patients [3,7]. These examination findings have low sensitivity but high specificity, thus they can guide further investigation [11], as concluded by Chen et al.'s study [12], in which most of the patients had abnormal characteristics of four limbs blood pressure (4LBP) that helped them to diagnose the disease by performing non-invasive primary screening and complete assessment of the patients. Similarly, in our patient, an initial physical examination demonstrated that the right arm was cold and pulseless, with undetectable blood pressure, while the left arm and both the lower limbs had detectable pulses and normal blood pressures, which raised the suspicion of Takayasu’s arteritis.

Along with physical examination, imaging studies are required to diagnose Takayasu’s arteritis. In our patient, MRA was used, which has an accuracy of 85%, sensitivity of 90%, and specificity of 80% in detecting the lesions of the aortic arch and its branches, and an accuracy of 90%, sensitivity of 98% and specificity of 90% in detecting the lesions of the abdominal aorta and its branches [13]. Positron emission tomography/computed tomography (PET/CT) was not done due to unavailability and cost issues. It is a useful imaging modality to detect early lesions and active inflammation in the vessels [14]. 

Diagnosis is based on clinical features and angiographic findings. The American College of Rheumatology criteria 1990 is commonly used for the diagnosis of Takayasu’s Arteritis [6]: age of onset less than 40 years, claudication of extremities, decreased brachial artery pressure, blood pressure differences of more than 10 mm/Hg between the arms, bruits over subclavian arteries and aorta, and aortogram abnormalities. The presence of three or more of these six criteria demonstrated a sensitivity of 90.5% and a specificity of 97.8% [6]. In our patient, four out of these six criteria were present and so the diagnosis of Takayasu’s arteritis was confirmed.

The differential diagnosis includes giant cell arteritis, infections like syphilis and tuberculosis, and connective tissue disorders like systemic lupus erythematosus (SLE), rheumatoid arthritis, spondyloarthropathies, all of which can lead to large vessel disease. Therefore, epidemiological and demographic features complement the clinical, laboratory, and imaging findings to diagnose Takayasu’s arteritis [15].

The goal of the treatment is immunosuppression and systemic steroids are the first line of management, but they have a high rate of relapse and are commonly associated with adverse effects [1]. Therefore, in most cases, nonsteroidal immunosuppressive agents like methotrexate and cyclophosphamide are required for long-term disease control [1]. Since our patient’s clinical and laboratory parameters were improving with corticosteroids alone, other immunosuppressants were not considered. Mechanical thrombectomy is indicated for patients who present within 24 hours of the onset of acute ischemic stroke involving anterior circulation [16], but it was not considered for our patient due to unavailability issues. Revascularization surgeries such as percutaneous transluminal angioplasty and open surgical bypass grafting are also recommended for aneurysmal disease or severe stenosis of the major arteries such as cerebral or coronary arteries [17], but it was not done in our patient due to financial constraints.

Stroke in young patients with Takayasu’s arteritis can be debilitating to their physical and mental health and, sometimes, it can also be life-threatening. As compared to elderly patients with stroke, young patients are disabled for a longer duration of their life, while becoming dependent during their most productive years, which can have a greater impact on their mental and socioeconomic status [18]. So early diagnosis, treatment, and monitoring are of utmost importance to control the disease activity and prevent life-threatening complications.

## Conclusions

In conclusion, young patients, who present with stroke, should be properly evaluated for Takayasu’s arteritis. For its diagnosis, examination of all the peripheral pulses and recording of the blood pressure in all four limbs is essential. It also provides guidance for further investigations like angiography. Early identification of the disease is important, as continuous medical management, along with timely interventions, will halt the inflammation and its progression to the permanent stenotic stage, preventing end-organ damage, and thereby, leading to better clinical outcomes.
